# Motivational variations in fitness: a population study of exercise modalities, gender and relationship status

**DOI:** 10.3389/fpsyg.2024.1377947

**Published:** 2024-03-21

**Authors:** Vojko Vuckovic, Sasa Duric

**Affiliations:** ^1^Faculty of Sport, University of Ljubljana, Ljubljana, Slovenia; ^2^Liberal Arts Department, American University of the Middle East, Egaila, Kuwait

**Keywords:** motivation, group exercise, personal trainer, recreation, EMI-2 questionnaire, Slovenia

## Abstract

**Introduction:**

Motivation plays a crucial role in determining whether or not a person adheres to an exercise program. The present study aimed to determine the motivational differences between people exercising in fitness alone, in groups/aerobics and with a personal trainer by gender and relationship status.

**Methods:**

The Exercise Motivations Inventory-2 (EMI-2) questionnaire was completed by 830 users of 20 largest fitness centers in Slovenia.

**Results:**

The Kruskal-Wallis test followed by a Dunn post-hoc test revealed that health-related motives such as ill-health avoidance were most frequently associated with exercising with a personal trainer compared to other exercise modalities, especially among females (*p* = 0.032, *M* = 4.88) and people in a relationship (*p* = 0.020, *M* = 5.18). On the other hand, intrinsic motivations such as enjoyment and stress management were mostly associated with exercising alone (*p* = 0.002, *M* = 4.98 and *p* = 0.021, *M* = 4.68, respectively). These results were also transferred to females and to some extent to people in a relationship (for enjoyment only). It is expected that intrinsic motivation is related to sustained exercise behavior.

**Discussion:**

Future studies could implement a longitudinal design to test this statement and examine the proposed relationships over a longer period to better understand whether there may be causal relationships between motivation and different exercise modalities depending on different characteristics of participants.

## 1 Introduction

Physical inactivity remains a significant problem that negatively impacts the physical (Haskell et al., 2007) and mental health of adults (Spalter et al., 2015; Kajtna and Vučković, 2022). One of the fastest growing sectors in physical activity and exercise is the fitness industry (Rodriguez, 2019; Yi et al., 2021), which is particularly popular among young people (Ong et al., 2021). There are around 185 million members and 210,000 clubs worldwide (Rothmann, 2022). In a study conducted by Fernandez (2022), gym members had a lower prevalence of physical inactivity and a higher prevalence of vigorous physical activity than the general population, regardless of age and gender. However, less than 40% of gym members exercise regularly and the dropout rate is high (Sperandei et al., 2016; Kopp et al., 2020; Rand et al., 2020; Gjestvang et al., 2021).

It is known that motivation plays a crucial role in determining whether or not a person adheres to an exercise program (Teixeira et al., 2012). Nevertheless, research on the exercise behaviors of fitness club members is quantitatively and qualitatively limited (Middelkamp and Steenbergen, 2015; Gjestvang et al., 2021). Frederick and Ryan (1993) found that people who engage in sports are driven by interest/enjoyment and competence motivation, whereas people who engage in fitness activities are driven by body-related motivation. Kilpatrick et al. (2005) also distinguish that intrinsic motives such as enjoyment and challenge are responsible for engaging in sport, while motivation for fitness training is more extrinsic and focuses on appearance, weight and stress management. Exercise behavior is determined more by health and fitness motives and also by appearance/weight concerns than by participation in sport. In contrast, social engagement and enjoyment motives were found to be less associated with fitness training, but more motivating for sport participation (Cagas et al., 2015).

Regarding the different types of activities, it was demonstrated that the strongest discriminators were affiliation in team sports, enjoyment in individual racing sport and bowls players, mastery in racquet sports, psychological state in fitness exercisers, and competition/ego in martial artists (Molanorouzi et al., 2015). The three most commonly cited motives for participants in fitness training and recreational activities were strength and endurance, weight management and stress management (Ball et al., 2014; Rodrigues et al., 2022). Rodrigues et al. (2022) found differences between water activities and group fitness classes and cardio/strength activities. Few studies have been conducted with participants involved in extreme conditioning program training. Fisher et al. (2017) suggest that these participants were more likely to report higher scores for intrinsic motives such as enjoyment, challenge, and affiliation, while personal training clients reported higher scores for health-related motives such as positive health, ill-health avoidance, and weight management. The same authors suggest that individuals who exercise one-to-one with a personal trainer have higher health-related motives (e.g., positive health, health pressures, and ill-health avoidance). Similar results were found by Marin et al. (2018), where participants involved in extreme conditioning program training had higher levels of enjoyment, stress management, social recognition, affiliation, competition and weight management. Conversely, resistance training participants indicated a higher motive for appearance.

Although some previous studies have found differences in motivation to participate in sport and fitness training (Whaley, 2003; Kilpatrick et al., 2005), there is a gap in the literature when it comes to motivation to participate in the latter (Ball et al., 2014). Recent studies also agree that future studies should analyze the motivational differences between different exercise activities in more detail (Rodrigues et al., 2022). In a study by Tsitskari et al. (2017), exercise motivation was used to segment gym-goers and conduct further analyses. However, there are no studies in which the motivation to participate in different types of exercise in fitness centers was investigated.

Very few studies compared extreme conditioning program training with group fitness exercise, training alone and also training with a personal trainer using the Exercise Motivations Inventory-2 (EMI-2) questionnaire (Fisher et al., 2017). However, the design of the aforementioned study has some weaknesses. Firstly, the authors obtained the responses via social media. The fact that the authors were not personally present in the fitness centers could have an impact on the honesty of the participants and thus on the reliability and validity of the analyzed data. Although some authors claim that the presence of the researcher has no effect on performance (Wood et al., 2006), there are some other studies that show that the virtual presence of the researcher had a significant positive effect in preventing careless responding (Ward and Pond, 2015). Secondly, the criterion in the mentioned study was that all participants had been exercising for more than 6 months. The study by Maltby and Day (2001) has shown that this is a very important factor in determining motivation to exercise, so we believe that this could bias the results obtained. We believe that all participants should be assessed regardless of their training experience, as extrinsic motivation plays a decisive role, especially in the initial phases of training, while intrinsic motives are crucial for progress in later phases (Dacey et al., 2008; Jones et al., 2020). Another study by Marin et al. (2018) used the EMI-2 questionnaire to assess motivational differences between resistance training and extreme conditioning program training. However, they used a similar approach by creating an online questionnaire and posting it on Facebook, where it remained for 2 months. In a study from China (Rahman et al., 2019), the authors also used the EMI-2 questionnaire to assess the participants’ motivation for physical activity for three different group activities: fitness training, sports, and recreational and cultural activities. The drawback of this study was that the participants were predominantly middle-aged and older, mainly 55–64-year-olds and 65–74-year-olds (the inclusion criterion was age 35+). If we truly want to understand the motivational structure of exercisers, we should examine the entire age span and include youth as well. One of the reasons for this is that many studies have shown that the motivation for exercise varies according to age group (Biddle et al., 2003; Trujillo et al., 2004; Caglar et al., 2009; Brunet and Sabiston, 2011; Egli et al., 2011; Molanorouzi et al., 2015; Jones et al., 2020; Box et al., 2021; Grajek et al., 2021; Gut et al., 2022; Rodrigues et al., 2022), so that the assessment of motivational structures between different exercise modalities would lead to biased results in older adults. Another advantage of including young adults in the study is the fact that they can be guided toward a more active lifestyle, if this is not already the case. Furthermore, there is ample evidence that the level of physical activity in adolescents continues into adulthood (Telama et al., 2005; Telama, 2009). It is also worth noting that previous studies have shown that motivations related to weight control and physical appearance were prevalent in women, whereas all aspects related to competition were prevalent in men (Kilpatrick et al., 2005; Pauline, 2013; Vuckovic et al., 2023). Furthermore, to our knowledge, there are no studies that consider relationship status when examining exercise motivation.

For the abovementioned reasons, we designed a study to investigate the differences in motivation for exercise between participants engaged in different fitness training modalities. We applied a direct and holistic approach that considered the gender and relationship status of the participants, thus significantly improving the methodological approach used previously. More specifically, the aim of the present study was to determine the differences in motivational structure between fitness, aerobics, and personal trainer adult clients.

## 2 Materials and methods

### 2.1 Participants

We collected data from members of 20 fitness centers in 9 major Slovenian cities (4 from the eastern region and 5 from the western region). The questionnaire was distributed to 2,060 participants. After removing incomplete answers (including attention test), the final number of fully and correctly completed questionnaires for further processing amounted to 830 questionnaires, which corresponds to a response rate of 40.29%.

Table 1 shows the demographic characteristics of the fitness center users.

**TABLE 1:** Sample characteristics.

	Percentage	n
**Gender**		
Male	57%	471
Female	43%	359
**Age (years)**		
Mean	27.3	
Standard deviation	11.3	
Range	18–70	
**Relationship status**		
Single	49%	405
In relationship	51%	425
**Education**		
Elementary school degree	2%	18
Secondary school degree	48%	402
High school degree	19%	154
College degree	26%	215
Master’s or doctoral degree	5%	41
**Occupation**		
Student	46%	383
Unemployed	2%	17
Corporate employee	40%	329
Self-employee	11%	90
Retiree	1%	11

### 2.2 Procedures

It is worth noting that all the major fitness centers in Slovenia that we have selected have relatively similar characteristics in terms of facilities, equipment, programs offered, membership conditions and prices. After the participants completed their workout and left the respective centers, we approached them with tablet computers. To encourage their participation, we offered them protein bars and asked them to complete the questionnaire. Before handing over the questionnaire, the procedure was explained in details to address any potential concerns or uncertainties the participants might have. All questionnaires were distributed from Monday to Sunday, in the morning, afternoon and evening. Participants signed a consent form before completing the questionnaires via an online cloud platform specifically designed for survey purposes.^1^ This study was conducted in accordance with the Declaration of Helsinki. All participants gave written informed consent before participating in the study, and the Ethics Committee of the University of Ljubljana granted ethical approval for data collection (No. 2021-19).

### 2.3 Instruments

The Exercise Motivations Inventory-2 (EMI-2), developed by Markland and Ingledew (1997) was used to assess the exercise motivation of fitness center members. The EMI-2 scale consists of 51 items and each item is measured on a 6-point Likert scale from zero (does not apply to me at all) to five (applies to me very much), with higher scores indicating higher motivation to exercise. These items form 14 subscales, including: Affiliation, Appearance, Challenge, Competition, Enjoyment, Health Pressure, Disease Prevention, Agility, Positive Health, Revitalization, Social Recognition, Strength and Endurance, Stress Management, and Weight Management. Each subscale is determined by calculating the average of 3 to 4 appropriate items based on the EMI-2 scale scoring key. The EMI-2 is a factorially valid mean of assessing a wide range of motives for participation in sporting activities in adult men and women and is suitable for both athletes and non-athletes (Markland and Ingledew, 1997). The EMI-2 has already been used in the Slovenian population, with Cronbach’s alpha ranging from 0.71 to 0.91 (Vuckovic et al., 2023). However, we confirmed the reliability of the 51 EMI-2 items in this study by measuring the Cronbach’s Alpha coefficient (α = 0.801) and the 14 scales (α ranged from 0.665 to 0.903). In addition, the attention test questions were interspersed in the questionnaire to further increase the reliability and validity of the assessment. All participants who did not answer the attention test questions correctly were excluded from the study. In addition, participants answered questions about their personal life and the type of training they were participating in. For reasons of ecological validity, participants who took part in more than one type of training were also excluded from the study (see section “2.1 Participants”).

### 2.4 Statistical analysis

The Cronbach’s Alpha test was used to test the reliability of the EMI-2 scales. The mean scores of the 14 motivational scales were used as dependent variables, while the type of participant engagement (fitness, group training or personal trainer) and participant characteristics such as gender (males vs. females) and relationship status (single vs. in a relationship/married) were used as independent variables. Due to the ordinal and nominal nature of the data, the non-parametric Kruskal-Wallis test was used in this study. In the case of significant differences, the Dunn post-hoc test was used for pairwise comparisons. For each significant difference, the effect size was also reported as Pearson’s r, with r values of 0.10, 0.30, and 0.50 representing the thresholds for small, medium and large effects respectively (Cohen, 1988). Descriptive statistics were presented as means, standard deviations, and χ^2^. All statistical tests were analyzed using the RStudio software (RStudio; Posit, PBC, Vienna, Austria). The alpha level was set at 0.05.

## 3 Results

Table 2 contains descriptive statistics on the observed variables and the results of the Kruskal-Wallis test for the whole sample.

**TABLE 2:** Motivations of fitness center users by type of exercise.

Motive	Exercise	n	M	SD	χ^2^	p
Stress management	Fitness	686	4.68	1.13	7.75	0.021*
	Group exercise	96	4.53	1.20		
	Personal trainer	48	4.27	1.16		
Revitalisation	Fitness	686	5.11	0.96	1.11	0.573
	Group exercise	96	5.11	1.08		
	Personal trainer	48	4.99	1.11		
Enjoyment	Fitness	686	4.98	0.99	12.89	0.002**
	Group exercise	96	4.70	1.15		
	Personal trainer	48	4.45	1.28		
Challenge	Fitness	686	4.48	1.15	7.64	0.022*
	Group exercise	96	4.11	1.27		
	Personal trainer	48	4.35	1.26		
Social recognition	Fitness	686	3.09	1.41	7.38	0.024*
	Group exercise	96	2.69	1.44		
	Personal trainer	48	2.92	1.36		
Affiliation	Fitness	686	3.74	1.40	11.48	0.003**
	Group exercise	96	4.15	1.46		
	Personal trainer	48	3.41	1.40		
Competition	Fitness	686	3.39	1.58	11.45	0.003**
	Group exercise	96	2.80	1.54		
	Personal trainer	48	3.21	1.67		
Health pressures	Fitness	686	2.62	1.33	3.11	0.211
	Group exercise	96	2.78	1.45		
	Personal trainer	48	2.89	1.25		
Ill-health avoidance	Fitness	686	4.51	1.22	10.39	0.006**
	Group exercise	96	4.80	1.25		
	Personal trainer	48	4.88	1.15		
Positive health	Fitness	686	5.26	0.86	2.22	0.330
	Group exercise	96	5.32	0.95		
	Personal trainer	48	5.25	1.07		
Weight management	Fitness	686	4.32	1.25	1.80	0.406
	Group exercise	96	4.46	1.30		
	Personal trainer	48	4.25	1.23		
Appearance	Fitness	686	4.71	0.99	11.38	0.003**
	Group exercise	96	4.37	1.20		
	Personal trainer	48	4.28	1.21		
Strength and endurance	Fitness	686	5.29	0.78	4.09	0.130
	Group exercise	96	5.08	1.00		
	Personal trainer	48	5.00	1.18		
Nimbleness	Fitness	686	4.73	1.13	7.37	0.025*
	Group exercise	96	4.94	1.06		
	Personal trainer	48	5.01	1.19		

M, mean; SD, standard deviation; p, *p*-value;

*p < 0.05;

**p < 0.01.

Post-hoc tests revealed that individuals who exercise alone in the fitness center are significantly more motivated by stress management than personal trainer clients (*p* = 0.008; *r* = 0.097). They are also more motivated by enjoyment compared to group exercise (*p* = 0.025; *r* = 0.080) and personal trainer clients (*p* = 0.003; *r* = 0.110). Similarly, fitness center users are significantly more motivated by challenge (*p* = 0.006; *r* = 0.098) and social recognition (*p* = 0.008; *r* = 0.096) than group exercise users. Users who participate in group classes exercise significantly more out of a sense of belonging than users who exercise in a fitness (*p* = 0.004; *r* = 0.100) or with a personal trainer (*p* = 0.002; *r* = 0.250). We also found that fitness center users are significantly more motivated by competition (*p* = 0.001; *r* = 0.120) than those who attend group classes. We also demonstrated that fitness center users are more interested in their appearance than those attending group classes (*p* = 0.010; *r* = 0.092) or training with a personal trainer (*p* = 0.016; *r* = 0.089), but exercise less because for ill-health avoidance reasons than those attending group (*p* = 0.010; *r* = 0.092) and personal training (*p* = 0.030; *r* = 0.080). Finally, our results show that personal training clients exercise much more for nimbleness reasons than fitness participants (*p* = 0.032; *r* = 0.079).

Table 3 shows the different motivations for exercise between the genders.

**TABLE 3:** Mean ranking scores on exercise motivation between male and female participants.

		Males					Females				
**Motive**	**Exercise**	**n**	**M**	**SD**	**χ^2^**	**p**	**n**	**M**	**SD**	**χ^2^**	**p**
Stress management	Fitness	433	4.62	1.16	2.68	0.262	253	4.79	1.06	6.90	0.032*
	Group exercise	17	4.49	1.20			79	4.54	1.21		
	Personal trainer	21	4.32	1.04			27	4.23	1.27		
Revitalisation	Fitness	433	5.06	0.98	1.38	0.503	253	5.18	0.91	0.86	0.649
	Group exercise	17	4.75	1.21			79	5.19	1.04		
	Personal trainer	21	4.94	0.98			27	5.02	1.22		
Enjoyment	Fitness	433	5.00	0.99	2.76	0.252	253	4.95	0.99	8.51	0.014*
	Group exercise	17	4.72	1.18			79	4.69	1.15		
	Personal trainer	21	4.69	1.05			27	4.26	1.42		
Challenge	Fitness	433	4.50	1.16	0.53	0.765	253	4.45	1.13	6.87	0.032*
	Group exercise	17	4.41	1.22			79	4.04	1.27		
	Personal trainer	21	4.70	1.09			27	4.08	1.33		
Social recognition	Fitness	433	3.25	1.45	0.17	0.917	253	2.82	1.28	3.40	0.183
	Group exercise	17	3.25	1.55			79	2.57	1.39		
	Personal trainer	21	3.36	1.38			27	2.57	1.27		
Affiliation	Fitness	433	3.81	1.39	4.85	0.088	253	3.62	1.39	11.25	0.004**
	Group exercise	17	4.51	1.30			79	4.07	1.49		
	Personal trainer	21	3.82	1.21			27	3.08	1.47		
Competition	Fitness	433	3.63	1.57	2.14	0.342	253	2.98	1.51	5.49	0.064
	Group exercise	17	3.78	1.49			79	2.59	1.48		
	Personal trainer	21	4.12	1.53			27	2.50	1.43		
Health pressures	Fitness	433	2.65	1.37	5.81	0.055	253	2.56	1.25	2.27	0.321
	Group exercise	17	3.45	1.45			79	2.63	1.42		
	Personal trainer	21	2.83	1.22			27	2.94	1.29		
Ill-health avoidance	Fitness	433	4.47	1.27	2.17	0.337	253	4.57	1.14	6.88	0.032*
	Group exercise	17	4.63	1.52			79	4.84	1.19		
	Personal trainer	21	4.89	0.90			27	4.88	1.33		
Positive health	Fitness	433	5.20	0.88	0.73	0.695	253	5.36	0.82	0.29	0.867
	Group exercise	17	5.10	1.19			79	5.37	0.89		
	Personal trainer	21	5.38	0.73			27	5.15	1.28		
Weight management	Fitness	433	4.22	1.28	0.30	0.860	253	4.50	1.18	0.36	0.836
	Group exercise	17	4.10	1.52			79	4.53	1.24		
	Personal trainer	21	4.20	0.86			27	4.29	1.48		
Appearance	Fitness	433	4.70	0.99	6.62	0.036*	253	4.71	1.00	6.04	0.049*
	Group exercise	17	3.96	1.45			79	4.46	1.14		
	Personal trainer	21	4.38	1.09			27	4.20	1.31		
Strength and endurance	Fitness	433	5.33	0.75	1.25	0.536	253	5.22	0.82	1.05	0.593
	Group exercise	17	4.96	1.19			79	5.11	0.96		
	Personal trainer	21	5.10	0.98			27	4.93	1.33		
Nimbleness	Fitness	433	4.70	1.17	2.97	0.227	253	4.77	1.06	3.75	0.154
	Group exercise	17	4.90	1.19			79	4.95	1.03		
	Personal trainer	21	5.10	0.94			27	4.94	1.37		

M, mean; SD, standard deviation; p, *p*-value;

*p < 0.05;

**p < 0.01.

Using post-hoc tests, we found that affiliation (*p* = 0.028; *r* = 0.103) and health pressures (*p* = 0.020; *r* = 0.109) motivates males much more to go to group exercise classes than to fitness training. Completely opposite results were found for the motive appearance – males who exercise because of this go to fitness and do not prefer group exercise (*p* = 0.029; *r* = 0.103).

Our results also demonstrate that female fitness center users who exercise alone are motivated by stress management (*p* = 0.019; *r* = 0.140) or enjoyment (*p* = 0.009; *r* = 0.156) are significantly more likely to work out alone in fitness than to go to a personal trainer. Those who exercise for challenge (*p* = 0.015; *r* = 0.134) and competition (*p* = 0.049; *r* = 0.108) are also more likely to exercise in a fitness than in a group, in contrast to those who are motivated with ill-health avoidance (*p* = 0.031; *r* = 0.119). Finally, women who are motivated by affiliation are more likely to participate in group training than in fitness training alone (*p* = 0.010; *r* = 0.142) or with personal trainer (*p* = 0.002; *r* = 0.297).

Table 4 shows how the exercise motives of the single participants and the participants living in a relationship differ in relation to the type of training.

**TABLE 4:** Mean ranking scores on exercise motivation between single participants and participants in a relationship.

		Singles					In a relationship/married				
**Motive**	**Exercise**	**n**	**M**	**SD**	**χ^2^**	**p**	**n**	**M**	**SD**	**χ^2^**	**p**
Stress management	Fitness	360	4.70	1.09	5.68	0.059	326	4.66	1.17	3.84	0.146
	Group exercise	33	4.71	1.10			63	4.44	1.25		
	Personal Trainer	14	3.84	1.44			34	4.45	1.00		
Revitalisation	Fitness	360	5.07	0.95	2.64	0.270	326	5.15	0.96	0.17	0.918
	Group exercise	33	5.10	1.08			63	5.12	1.09		
	Personal Trainer	14	4.52	1.43			34	5.18	0.90		
Enjoyment	Fitness	360	5.03	0.99	4.22	0.121	326	4.92	1.00	6.93	0.031*
	Group exercise	33	4.74	1.10			63	4.67	1.18		
	Personal Trainer	14	4.29	1.73			34	4.51	1.06		
Challenge	Fitness	360	4.59	1.08	2.93	0.232	326	4.36	1.20	3.48	0.175
	Group exercise	33	4.30	1.23			63	4.03	1.29		
	Personal Trainer	14	4.13	1.66			34	4.45	1.06		
Social recognition	Fitness	360	3.22	1.36	2.39	0.303	326	2.95	1.45	5.84	0.054
	Group exercise	33	3.06	1.46			63	2.49	1.39		
	Personal Trainer	14	2.70	1.39			34	3.01	1.36		
Affiliation	Fitness	360	3.90	1.35	6.96	0.031*	326	3.58	1.43	6.19	0.045*
	Group exercise	33	4.44	1.35			63	4.00	1.50		
	Personal Trainer	14	3.48	1.43			34	3.38	1.41		
Competition	Fitness	360	3.60	1.55	3.09	0.214	326	3.16	1.58	8.30	0.016*
	Group exercise	33	3.28	1.61			63	2.56	1.45		
	Personal Trainer	14	2.98	1.78			34	3.30	1.65		
Health pressures	Fitness	360	2.56	1.30	0.36	0.835	326	2.67	1.36	3.20	0.202
	Group exercise	33	2.51	1.41			63	2.92	1.46		
	Personal Trainer	14	2.60	0.80			34	3.01	1.39		
Ill-health avoidance	Fitness	360	4.34	1.25	0.65	0.722	326	4.69	1.17	7.85	0.020*
	Group exercise	33	4.46	1.38			63	4.97	1.15		
	Personal trainer	14	4.17	1.52			34	5.18	0.82		
Positive health	Fitness	360	5.22	0.88	0.03	0.984	326	5.31	0.84	2.62	0.270
	Group exercise	33	5.12	1.09			63	5.43	0.85		
	Personal trainer	14	4.79	1.64			34	5.44	0.68		
Weight management	Fitness	360	4.29	1.28	2.34	0.310	326	4.36	1.22	1.53	0.464
	Group exercise	33	4.36	1.22			63	4.51	1.34		
	Personal Trainer	14	3.80	1.26			34	4.43	1.19		
Appearance	Fitness	360	4.78	0.89	9.40	0.009**	326	4.63	1.09	3.72	0.156
	Group exercise	33	4.43	1.21			63	4.34	1.21		
	Personal trainer	14	3.91	1.26			34	4.43	1.18		
Strength and endurance	Fitness	360	5.33	0.74	1.55	0.461	326	5.30	0.82	1.91	0.385
	Group exercise	33	5.13	0.98			63	5.06	1.01		
	Personal trainer	14	4.64	1.73			34	5.15	0.85		
Nimbleness	Fitness	360	4.68	1.11	0.96	0.619	326	4.78	1.14	5.05	0.080
	Group exercise	33	4.87	0.96			63	4.98	1.11		
	Personal trainer	14	4.60	1.73			34	5.18	0.86		

M, mean; SD, standard deviation; p, p-value;

*p < 0.05;

**p < 0.01.

Post-hoc tests showed that singles prefer fitness (*p* = 0.017; *r* = 0.123) or group exercise (*p* = 0.041; *r* = 0.299) rather than training with a personal trainer to cope with everyday stress. They also exercise much more in a group than in a fitness (*p* = 0.018; *r* = 0.120) or with a personal trainer (*p* = 0.026; *r* = 0.324) because of affiliation. Singles also exercise more alone in a fitness than with a personal trainer because of their appearance (*p* = 0.007; *r* = 0.141).

Members of fitness centers who are in a relationship or married enjoy fitness training more than training with a personal trainer (*p* = 0.017; *r* = 0.126). They also exercise more in the fitness than in groups because they want to be socially recognized (*p* = 0.020; *r* = 0.118). In addition, those who exercise because of affiliation, exercise more in groups than in the fitness (*p* = 0.023; *r* = 0.115) and with personal trainer (*p* = 0.037; *r* = 0.212). And those who exercise for competitive reasons exercise less in groups and more in the fitness area (*p* = 0.006; *r* = 0.140) and with a personal trainer (*p* = 0.028; *r* = 0.223). Finally, avoiding illness is a motive that is more common among users of group exercise (*p* = 0.039; *r* = 0.105) and personal trainer clients (*p* = 0.033; *r* = 0.113) than among users who only exercise in the fitness area.

## 4 Discussion

The aim of the study was to determine the motivational differences between people who train in fitness alone, in groups/aerobics, and those who train with a personal trainer. We also wanted to investigate whether the motivational structure of the participants differs according to gender and relationship status.

It is important to note that our analysis revealed no significant differences in the motivation scales for revitalisation, weight management, and strength and endurance, regardless of exercise type, gender, or relationship status. In the past, stress management has been shown to be a better motivation for exercise than sport activities (Kilpatrick et al., 2005; Ball et al., 2014), especially for extreme conditioning program training participants (Fisher et al., 2017; Marin et al., 2018). The results of our study show that the motive of coping with stress is more likely to be found among regular fitness center users than among group exercise and personal trainer clients. This was particularly pronounced in singles and females. We can only speculate about the reasons for the obtained results. One of the reasons could be that fitness center users prefer to train alone to cope with the stress rather than focusing on the personal trainer. Sometimes instructors can cause additional stress by requiring their practitioners to stay focused and follow instructions, especially if having a highly committed coach-athlete relationship (Nicholls et al., 2016). Another explanation could be that single people, especially females, minimize their approachability by having the personal trainer work with them. It is known that one of the reasons of going to fitness is socialization (Eriş et al., 2018). It is also known that physical activity can trigger the release of endorphins, which are natural mood boosters, and it can also help to lower levels of stress hormones such as cortisol (Habibzadeh, 2015). Women tend to have higher stress levels due to a variety of factors (Matud, 2004), which may make stress management a more compelling motivation. Finally, we can speculate that single men probably do not prefer to show their weaknesses by having someone tell them what and how to do in front of other people, especially potential “relationship candidates,” which is why they do not seek out personal trainer sessions but prefer to work out alone at the gym.

A strong motive for participants in sports (Kilpatrick et al., 2005) such as individual racing (Molanorouzi et al., 2015) and extreme conditioning program training (Fisher et al., 2017; Marin et al., 2018) is to have fun. The results of our study show that enjoyment is a very important motivating factor for women and people in a relationship to exercise alone. If we consider the previously mentioned factor of approachability for females, we could assume that people who are in a relationship may simply want to enjoy time to themselves without having to deal with anyone, including personal trainer. In addition, individuals who pursue an independent fitness routine may place more emphasis on having fun because they have more autonomy in choosing the activities they really like. They can choose exercises that suit their personal interests and bring them pleasure. Personal trainers or group training programmes, on the other hand, may take a more structured approach that focuses less on individual enjoyment.

Another important reason for participating in an independent fitness routine is the challenge, especially for females. This can be explained by the fact that participants in group exercise classes perform the same exercises, which makes them less challenging. For some, the personal challenge of setting and achieving fitness goals for themselves is a source of recognition and self-satisfaction. They enjoy the sense of fulfilment that comes from overcoming individual challenges. A study by Rodrigues et al. (2022) has shown that group exercise is also known to be less challenging than some individual water activities.

People who are intrinsically motivated are more likely to participate in activities because they enjoy them or feel challenged. By contrast, in personal training or group exercise, extrinsic motives such as external goals or expectations may play more of a role, so the enjoyment or challenge may play a secondary role. Challenge and enjoyment are known to be intrinsic motives (Markland et al., 1992; Frederick and Ryan, 1993; Markland and Ingledew, 1997; Maltby and Day, 2001; Egli et al., 2011; Fortier et al., 2012; Knowles et al., 2015) and generally have a positive influence on exercise participation (Dacey et al., 2008; Teixeira et al., 2012), while some authors also include stress management among the intrinsic motives (Markland and Ingledew, 1997; Maltby and Day, 2001).

Another important finding of our study is that participants, especially in a relationship/married, are more likely to participate in independent fitness activities rather than group activities due to social recognition. Previous studies suggest that social recognition is related to sport participation rather than exercise (Kilpatrick et al., 2005; Ball et al., 2014). Our findings contradict the results of a study by Fisher et al. (2017), which suggest that social recognition may be a motivating factor for participants in group exercise. However, in our case, the number of people who exercise individually is more than 7x times higher than the number of people who participate in group exercise. This means that people who exercise individually in the fitness have more contact with other people and automatically experience social recognition. On the other hand, the results of our study showed that group classes are very important for participants’ sense of belonging (affiliation) compared fitness and personal trainer sessions. This is in line with previous studies showing that aerobic exercise participants are primarily motivated by social-health factors (Laverie, 1998; Fisher et al., 2017). Other studies showed that affiliation is a very important motivating factor in sports participants (Kilpatrick et al., 2005; Ball et al., 2014), especially in team sports (Molanorouzi et al., 2015) or even extreme conditioning program training (Marin et al., 2018). In addition, a study from China showed that affiliation is very important when it comes to participating in cultural leisure activities (Rahman et al., 2019). It is common knowledge that people who want to belong somewhere join a group.

The analysis revealed that males are much more motivated by competition than females, which is consistent with previous studies (Morris et al., 1995; Morgan et al., 2003; Egli et al., 2011; Pauline, 2013; Boone and Brausch, 2016; Cho and Beck, 2016; Vučković et al., 2022; Vuckovic et al., 2023). However, there were no significant differences between the different exercise modalities. The results also showed that female fitness practitioners were more motivated by competition than female group exercisers. Previous research on this motive is inconclusive. In a study by Fisher et al. (2017), the authors claim that competition is more important in group training than when training alone or with a personal trainer. At the same time, Rodrigues et al. (2022) argue that the competitive motive is more pronounced in water-based activities than in group training. However, the results obtained could also be explained by the same reason previously given for the challenge - same group exercises are less demanding than individually tailored exercises, so the scope for competition is minimal.

It is known that health-related exercise motives are less associated with sport and more with exercise (Kilpatrick et al., 2005; Ball et al., 2014). Some studies have emphasized the importance of health motives specifically for aerobic exercise (Laverie, 1998) and others for training with a personal trainer (Fisher et al., 2017). The results of our study confirm both - that people who want to avoid poor health are more likely to exercise in a group and with a personal trainer than alone. This is in line with some previous studies, such as a study by Rodrigues et al. (2022), which suggests that health motives are a more important motivational factor for resistance training compared to various water activities. Interestingly, our further analysis showed that this is particularly true for participants who are in a relationship or married. As can be seen, in addition to affiliation, health was also an important motivational parameter for the decision to participate in group training.

It has been shown that appearance is more important for individual fitness users than for those who train in groups or with a personal trainer. Even more interesting is that this motivational factor is more important for single men. Individual training can be more time efficient. They can structure their workouts to fit their schedule, and this time efficiency can be especially important for their work-life balance. Previous studies have shown that physical appearance is more important for participants in exercise than for sport participants (Kilpatrick et al., 2005; Ball et al., 2014). However, the literature on this topic is inconsistent. A study by Marin et al. (2018) associates the motive of appearance with resistance training, while Fisher et al. (2017) argue that appearance is more important for individuals exercising with a personal trainer than for group, individual or extreme conditioning program training participants.

Finally, the results of our study showed that in addition to health-related motives, nimbleness was more important for participants who train with a personal trainer than for who train in fitness alone. This can be easily explained by the fact that personal trainer training sessions are usually conducted by highly qualified experts and are tailored to the specific needs, goals and fitness level of the clients.

### 4.1 Strengths and limitations

The main strength of our study is that the sample can be considered representative of the Slovenian population exercising in fitness centers. Our study included the 20 largest fitness centers in the country, which are evenly distributed throughout the country. For this reason, our results are significant and valuable for interpretation in the Slovenian population. Secondly, we were physically present during the assessment, so we are confident that the questionnaires were filled out by people who exercise regularly, so there is no room for misuse or misrepresentation. In addition, although we used the previously used reliable and valid EMI-2 questionnaire, to improve reliability and validity, we introduced “attention check” questions to ensure that the responses obtained were legitimate.

At the same time, some limitations of the study should be noted. Compared to regular fitness center members who exercised alone, a relatively small sample of participants attended group and personal trainer sessions. This finding is natural and was to be expected, but is still worth mentioning. One of the possible reasons for this could be that we surveyed the 20 largest fitness centers and the trend is toward exercising alone, while group and personal trainer sessions are more common in smaller gyms. General limitations also include the fact that this study was a cross-sectional study where data was collected all at once and there was no effective follow-up study. In addition, the intensity and frequency of exercise sessions were not recorded, although the participants exercised regularly. Furthermore, this study cannot be generalized globally due to the cultural and sociological characteristics of Slovenia. Future studies could introduce a longitudinal design to examine the proposed relationships at multiple time points over a longer period to better understand whether there may be causal relationships between motivation and different exercise modalities depending on different characteristics of the participants (gender, age, relationship status, etc.). The frequency and intensity of training as well as training experience should also be taken into account in future studies.

## 5 Conclusion

The results of our study showed that there are motivational differences between people who exercise in fitness alone, in groups/aerobics, and with a personal trainer, especially when gender and relationship status were taken into account.

Overall, fitness center members who exercised alone were motivated by stress management, enjoyment, challenge, social recognition, competition and appearance. Individuals who worked out in a group felt that affiliation was a very important reason for participating. People who exercised with a personal trainer did so primarily for reasons of disease prevention and nimbleness. These motivation scales were further translated to the participants according to gender and relationship status. Males exercised alone more than in groups, mainly because of their appearance. Females who exercised alone did so primarily for reasons of stress management, enjoyment, challenge and appearance compared to the other options. They also exercised with a personal trainer because they wanted to avoid illness, and in group classes for a sense of belonging (affiliation), just like single people. Individuals who were in a relationship, on the other hand, exercised alone for enjoyment and appearance, in a group for affiliation and with a personal trainer for competition and disease avoidance.

Health-related motives were most commonly associated with exercising with a personal trainer, especially among females and people in a relationship. Since nimbleness was also associated with personal training, this could be an indication of trust in the trainer as an expert in the field, especially when it comes to such serious matters as one’s health and physical performance. On the other hand, intrinsic motivations such as enjoyment and stress management were associated with exercising alone. These results were also transferred to females and, to a certain extent, to people in a relationship (enjoyment only). As mentioned earlier, this type of motivation is expected to be related to sustained exercise patterns (McDonough and Crocker, 2007; Markland and Tobin, 2010). The question is whether these fitness center members exercise the longest compared to others because they are inspired by intrinsic motivation. Future research is needed, preferably longitudinal.

## Data availability statement

The raw data supporting the conclusions of this article will be made available by the authors, without undue reservation upon request.

## Ethics statement

The studies involving humans were approved by the Ethics Committee of the University of Ljubljana. The studies were conducted in accordance with the local legislation and institutional requirements. The participants provided their written informed consent to participate in this study.

## Author contributions

VV: Conceptualization, Data curation, Formal analysis, Investigation, Methodology, Project administration, Resources, Software, Validation, Writing – original draft, Writing – review and editing. SD: Conceptualization, Data curation, Formal analysis, Investigation, Methodology, Supervision, Validation, Visualization, Writing – original draft, Writing – review and editing.

## Footnotes

1
www.1ka.si
